# Evaluation of liver kinase B1 downstream signaling expression in various breast cancers and relapse free survival after systemic chemotherapy treatment

**DOI:** 10.18632/oncotarget.27929

**Published:** 2021-05-25

**Authors:** Khoa Nguyen, Andrew Rivera, Madlin Alzoubi, Henri Wathieu, Shengli Dong, Hassan Yousefi, Margarite Matossian, Suresh Alahari, David Drewry, Matthew Burow, Bridgette Collins-Burow

**Affiliations:** ^1^Department of Medicine, Tulane University School of Medicine, New Orleans, LA, USA; ^2^Department of Biochemistry and Molecular Biology, LSUHSC School of Medicine, New Orleans, LA, USA; ^3^UNC Eshelman School of Pharmacy, Chapel Hill, NC, USA; ^*^These authors contributed equally to this work & are co-first authors

**Keywords:** LKB1, STK11, breast cancer, triple negative breast cancer, patient prognosis

## Abstract

LKB1-signaling has prominent roles in cancer development and metastasis. This report evaluates LKB1-signaling pathway gene expression associations with patient survival in overall breast cancer, specific subtypes, as well as pre- and post-chemotherapy. Subtypes analyzed were based on intrinsic molecular subtyping and traditional biomarker classifications. Intrinsic molecular subtypes included were Luminal-A, Luminal-B, HER2-enriched, and Basal-like. The biomarker subtypes assessed were Estrogen-Receptor Positive (ER+) and Negative (ER-), Wild-Type TP53 (WT-TP53) & Mutant-TP53, and Triple-Negative Breast Cancer (TNBC). Additionally, comparisons were made between these subtypes and breast cancer overall, and analyses between LKB1 signaling to patient survival before and after chemotherapy were made. We used the Kaplan-Meier Online Tool (KM Plotter) to correlate the relationship between mRNA expression of known LKB1 scaffolding proteins (CAB39 and LYK5), and downstream signaling targets (AMPK, MARK1, MARK2, MARK3, MARK4, NUAK1, NUAK2, PAK1, SIK1, SIK2, BRSK1, BRSK2, SNRK, and QSK), and patient survival across each subtype and treatment group. Our findings provide evidence that LKB1-signaling is associated with improved survival in overall breast cancer. Stratification into breast cancer subtypes show a more complicated relationship; NUAK2, for example, is correlated with improved survival in ER- but is worse in ER+ breast cancer. In evaluating the association of LKB1-signaling pathway expression with relapse free survival of varying breast cancer tumors exposed to chemotherapy or treatment-naive tumors, our data provides baseline knowledge for understanding the pathway dynamics that affect survival and therefore are linked to pathology. This establishes a foundation for studying LKB1 targets with the goal of identifying druggable targets.

## INTRODUCTION

Liver kinase B1 (LKB1), also known as STK11, is a ubiquitously expressed master serine/threonine kinase that has been demonstrated to have tumor suppressing activity. It plays integral roles in many cancer processes with functionally broad roles in controlling cell polarity, proliferation, differentiation, and metabolism [1, 2]. The role of LKB1 as a tumor suppressor was first recognized in Peutz-Jeghers syndrome patients where loss of function is correlated with an increased risk for developing cancers such as breast cancer [2–4]. While LKB1 is widely accepted as a tumor suppressor, some studies have demonstrated an oncogenic sequelae of LKB1 expression in specific cancer subsets [1]. Additionally, LKB1 mutations are commonly found in lung, cervical, hepatic, and other carcinomas. LKB1 is known to scaffold with 2 proteins (LYK5 and CAB39) to regulate 14 downstream kinases (AMPK, MARK1, MARK2, MARK3, MARK4, NUAK1, NUAK2, PAK1, SIK1, SIK2, BRSK1, BRSK2, SNRK, and QSK) [5]. Despite the current literature on LKB1 signaling in different diseases, its role in breast cancer remains understudied. This report seeks to fill that gap by identifying correlations between members of LKB1 signaling with patient outcomes in different breast cancer subtypes pre- and post-chemotherapy. Disease subtypes are grouped using intrinsic molecular subtypes (Luminal A, Luminal B, HER2-enriched, and Basal-like) as well as traditional biomarker-based classifications (ER+, ER-, Wild Type TP53, Mutant TP53, and TNBC). The ultimate goal of this report is to establish a baseline for clinical applications of targeted therapy, with results from intrinsic subtyping, while also establishing a foundation for basic science pursuits with results from biomarker-based grouping.

## MATERIALS AND METHODS

### The KM plotter online tool [6] was used to investigate the relationship between

A. mRNA expression of LKB1 signaling targets and patient survival across breast cancer subtypes Triple Negative Breast Cancer (TNBC), Estrogen Negative Receptor (ER-), Estrogen Positive Receptor (ER+), Wild Type P53 (WTP53), Mutant P53, Luminal A, Luminal B, HER2-enriched, and Basal-like (Supplementary Table 1).

B. Survival outcomes as a function of mRNA expression of LKB1 signaling pre- and post-systemic chemotherapy treatment in the previously mentioned breast cancer subtypes (Supplementary Table 2).

Parameters for cutoffs were *p* < 0.05 and Hazard Ratios that do not include 1.

Probe sets were selected based on the JetSet status, a scoring method for selecting the optimal probe [7]. For PAK1, analysis was performed using 2 probe sets (202161 and 226507) because both were tied in score in terms of primer specificity.

### Quantitative real time PCR to check LKB1 downstream baseline gene expression

Total RNA was isolated using the Quick-RNA Miniprep TM kit (Zymo Research, ZRC205705) according to manufacture protocol and the RNA was quantified and confirmed for quality using the Nanodrop spectrophotometer (ND-1000). cDNA synthesis was calculated and normalized based on a 1000 ng concentration and using the iScript cDNA synthesis kit (Bio-Rad 1708890) and amplified using the GeneAmp PCR system 9700.

#### Primer design

**Table:** 

Gene	Forward Oligo sequence (5′ to 3′)	Reverse Oligo sequence (5′ to 3′)
SIK1	CTCCGGGTGGGTTTTTACGAC	CTGCGTTTTGGTGACTCGATG
MARK1	GAGCGGGACACGGAAAATCAT	TGCTACTCGACTTGGTAGGCT
NUAK2	CGCCCAAGCCCCTAATGAAG	TCCCTCCGTATGTGCATCAGA
SIK2	AGACCACCCTCACATAATCAAAC	ATTTTCGCCTGGCTTCAGACT
SNRK	ATGGCAGGATTTAAGCGAGGG	GTTTAACCACGGCAAAATGGC
MARK3	ATTGCCAACGGTGAATGAACG	GCTGGTACGAGAGGTAACTTCTT
NAUK1	AAGGCACCTACGGCAAAGTC	GTCTGATGTGAACCATGTCTTGT
LKB1	TCCTTGTTTGCTACAGTTTCCTG	TCTGGCAGTATTGGGCATTTG
BRSK1	GAGGCCCGAAAGTTCTTCCG	CTCTGGACACGCATAATGGGG
MARK2	CACATTGGAAACTACCGGCTC	GGAGGAGTTCAGTTGAGTCTTGT
MARK4	AGGTTGCCATCAAGATTATCGAC	GATGCGGACTTCTCGGAACAG
BRSK2	AAAGCTGCACGACGTTTATGA	TGCGATGCGGATGTTGTTCT

#### Immunoblot and antibodies

Total protein was extracted from tumors using M-PER reagent from ThermoScientific (78505). Lysates were separated by gel electrophoresis on polyacrylamide gels, transferred to nitrocellulose membranes and detected by immunoblotting using an enhanced chemiluminescence system. The antibodies were obtained as follows: LKB1 (LKB1 (3047S), phospho-LKB1 (3482S), MARK3 (9311s), MARK4 (4834S), MARK2 (9118S), BRSK2 (5460S), ARK5/NUAK1 (4458S) from Cell Signaling and SIK1 (NBP1-82417), SIK2 (NB100-56458SS), SIK3 (NBP1-69207), AMPKα1 (NBP2-22127SS) from NOVUS, and SNRK (AB96762) from ABCAM, AMPK alpha2 (MAB2850) from R&D and Phospho-AMPKα 1,2 (44-1150G) from Invitrogen.

## RESULTS

### Association of LKB1 downstream kinase mRNA expression and patient survival in breast cancer subtypes using the Kaplan-Meier estimator

#### All breast cancer

Genes associated with increased survival were LKB1, AMPK, LYK5, MARK1, MARK2, NUAK2, PAK1 (both probe sets), SIK1, SIK2, BRSK1, BRSK2, SNRK, and QSK. The remaining genes did not make the statistical cutoff (Supplementary Table 1).

#### Luminal A

Genes associated with increased survival were LKB1, AMPK, LYK5, MARK1, MARK2, NUAK2, PAK1 (both probe sets), SIK1, SIK2, BRSK1, BRSK2, SNRK, and QSK. MARK4 was negatively associated with survival. The remaining genes did not make the statistical cutoff (Supplementary Table 1).

#### Luminal B

Genes associated with increased survival were LKB1, AMPK, LYK5, NUAK2, PAK1 (probe set 226507 but not 202161), SIK1, SIK2, BRSK1, SNRK, and QSK. Negatively associated survival genes were MARK3 and NUAK1. The remaining genes did not make the statistical cutoff (Supplementary Table 1).

#### HER2-enriched

Genes associated with increased survival were LYK5, MARK1, MARK4, NUAK2, PAK1 (both probe sets), BRSK1, BRSK2, and QSK. NUAK1 was negatively associated with survival. The remaining genes did not make the statistical cutoff (Supplementary Table 1).

#### Basal-like

Genes associated with increased survival were LYK5, MARK1, MARK2, NUAK2, PAK1 (both probe sets), SIK1, SIK2, BRSK1, BRSK2, and QSK. NUAK1 was negatively associated with survival. The remaining genes did not make the statistical cutoff (Supplementary Table 1).

#### ER -

Genes associated with increased survival were NUAK2, PAK1 (both probe sets), and BRSK1. Negatively associated survival genes are LKB1, CAB39, MARK1, MARK3, and NUAK1. The remaining genes did not make the statistical cutoff (Supplementary Table 2).

#### ER+

Genes associated with increased survival were MARK2, PAK1 (probe set 226507 but not 202161), SIK1, SNRK, and QSK. Negatively associated survival genes are MARK1, NUAK1, and NUAK2. The remaining genes did not make the statistical cutoff (Supplementary Table 2).

#### WT TP53

Genes associated with increased survival were AMPK, MARK3, and QSK. Negatively associated survival genes are LYK5, MARK2, MARK4, NUAK2, and PAK1 (probe set 202161 but not 226507). The remaining genes did not make the statistical cutoff (Supplementary Table 2).

#### Mutant TP53

Genes associated with increased survival were LKB1, MARK4, PAK1 (probe set 226507 but not 202161), SIK1, BRSK1, and QSK. Negatively associated survival genes were SIK2 and SNRK. The remaining genes did not make the statistical cutoff (Supplementary Table 2).

#### TNBC

Genes associated with increased survival were NUAK2, PAK1 (both probe sets), SIK2, and QSK. Negatively associated survival genes were MARK3, NUAK1, and SIK1. The remaining genes did not make the statistical cutoff (Supplementary Table 2).

### Association of LKB1 downstream kinase mRNA expression and patient survival in breast cancer subtypes pre- and post-systemic chemotherapy treatment using the Kaplan-Meier plotter database

In all breast cancer combined, high mRNA expression of SIK2 pre-chemotherapy correlated with reduced mortality. After chemotherapy, SIK2 expression was no longer significantly associated with patient survival. Survival did not differ significantly between high and low mRNA expression groups of AMPK, SNRK2, QSK pre- and post- chemo treatment (Table 1).

**Table 1:** Hazard ratios (HR) and associated confidence intervals (CI) comparing survival with and without systemic chemotherapy as a function of mRNA gene expression of select LKB1 downstream kinases in all breast cancer and intrinsic subtypes using the Kaplan–Meier estimator

		HR (CI)		*P* Value	
	Gene	No Treatment	Systemic Treatment	No Treatment	Systemic Treatment
All Breast Cancer	AMPK	**1.01 (0.82–1.25)**	**1.07 (0.9–1.28)**	0.0044	0.046
	NUAK1	1.23 (0.99–1.52)	**1.29 (1.08–1.54)**	0.061	0.0041
	SIK2	*0.32 (0.1–1.01)*	1.21 (0.78–1.87)	0.04	0.4
	BRSK2	0.81 (0.66–1.01)	*0.78 (0.65–0.92)*	0.058	0.0044
	SNRK2	**1.37 (1.11–1.69)**	**1.29 (1.08–1.53)**	0.0038	0.0046
	QSK	*0.67 (0.54–0.83)*	*0.7 (0.59–0.84)*	0.0002	0.000067
Luminal A	LKB1	***0 (0−Inf)***	***4.10E+09 (0−Inf)***	0.089	0.015
	AMPK	*0.62 (0.44–0.87)*	2.12 (0.88–5.11)	0.0054	0.087
	LYK5	1.24 (0.89–1.73)	**2.84 (1.17–6.87)**	0.2	0.016
	MARK2	*0.54 (0.39–0.75)*	*0.4 (0.17–0.97)*	0.00018	0.037
	MARK3	*0.68 (0.47–0.98)*	1.79 (0.76–4.21)	0.04	0.18
	NUAK1	**1.71 (1.14–2.55)**	1.57 (0.65–3.81)	0.0083	0.31
	NUAK2	*0.51 (0.34–0.75)*	*0.37 (0.16–0.9)*	0.00056	0.022
	PAK1 (202161)	*0.67 (0.47–0.94)*	0.57 (0.24–1.38)	0.018	0.21
	PAK1 (226507)	***0 (0−Inf)***	***0 (0−Inf)***	0.0047	0.001
	BRSK1	***0 (0−Inf)***	***1.03E+09 (0−Inf)***	0.027	0.2
	SNRK	*0.46 (0.31–0.68)*	0.51 (0.17–1.53)	0.00008	0.22
	QSK	*0.5 (0.36–0.69)*	0.67 (0.27–1.65)	0.000019	0.38
Luminal B	LKB1	-	*0.24 (0.05–1.09)*	-	0.045
	AMPK	*0.5 (0.33–0.75)*	*0.55 (0.32–0.95)*	0.00077	0.028
	CAB39	1.56 (0.97–2.5)	**1.94 (1.14–3.32)**	0.065	0.013
	MARK2	**1.49 (1.03–2.15)**	**2.43 (1.4–4.2)**	0.034	0.0011
	MARK4	*0.62 (0.41–0.93)*	0.66 (0.38–1.16)	0.02	0.14
	NUAK1	**1.83 (1.26–2.65)**	1.54 (0.9–2.63)	0.0012	0.11
	NUAK2	1.41 (0.95–2.1)	*0.46 (0.25–0.84)*	0.086	0.0098
	SIK1	*0.6 (0.41–0.88)*	**2.44 (1.23–4.84)**	0.0089	0.0087
	SIK2	-	**4.92 (0.88–27.51)**	-	0.046
	SNRK	*0.55 (0.37–0.81)*	0.75 (0.44–1.29)	0.0024	0.3
	QSK	*0.5 (0.33–0.77)*	1.38 (0.75–2.54)	0.0013	0.3
HER2-enriched	AMPK	**3.06 (1.07–8.75)**	1.97 (0.75–5.15)	0.028	0.16
	LYK5	***3.90E+08 (0−Inf)***	1.94 (0.94–4)	0.0041	0.068
	MARK1	-	**2.96 (1.04–8.4)**	-	0.033
	MARK2	1.99 (0.7–5.67)	**2.22 (1.05–4.67)**	0.19	0.032
	NUAK1	**6.07 (1.69–21.84)**	**3.44 (1.31–8.99)**	0.0017	0.0074
	NUAK2	*0.35 (0.12–1.02)*	0.47 (0.19–1.16)	0.044	0.093
	PAK1 (202161)	*0.27 (0.09–0.78)*	0.52 (0.21–1.28)	0.0093	0.15
	SNRK	1.84 (0.58–5.86)	**2.39 (1.03–5.58)**	0.3	0.037
	QSK	2.27 (0.8–6.5)	**2.17 (1.06–4.45)**	0.11	0.03
Basal-like	LKB1	2.22 (0.68–7.31)	**2.54 (1.3–4.97)**	0.18	0.0048
	LYK5	*0.61 (0.37–0.99)*	1.56 (0.85–2.88)	0.044	0.15
	MARK1	*0.21 (0.05–0.99)*	**1.88 (0.96–3.68)**	0.029	0.062
	MARK2	*0.61 (0.37–0.99)*	1.6 (0.98–2.64)	0.045	0.06
	MARK3	*0.46 (0.25–0.83)*	**2.32 (1.41–3.82)**	0.0082	0.00064
	NUAK1	1.37 (0.83–2.27)	**2.72 (1.5–4.93)**	0.22	0.00058
	SIK1	0.67 (0.41–1.1)	*0.46 (0.24–0.89)*	0.11	0.018
	BRSK1	1.55 (0.47–5.09)	*0.45 (0.23–0.89)*	0.46	0.018
	QSK	*0.57 (0.35–0.92)*	1.19 (0.72–1.97)	0.021	0.5

HR and associated CI colored in Italic indicate indicate positive survival effect, while those Bold indicates negative survival effect with statistical significance of *p* < 0.05; those Bold italic indicate statistical significance but a small sample size. Some genes are not shown either due to insufficient or non-significant data for both treatment conditions. (–) indicates insufficient data.

#### Luminal A

In Luminal A breast cancer, higher expression of MARK2, NUAK2, and PAK1 was significantly associated with improved survival in both pre- and post-chemotherapy groups. In the pre-chemotherapy group, increased NUAK1 expression was negatively associated with survival (Table 1).

#### Luminal B

In Luminal B breast cancer, AMPK expression was positively associated with survival in both pre- and post-chemotherapy groups, while MARK2 was negatively associated with survival in both groups. SIK1 expression was positively associated with survival pre-chemotherapy, but negatively associated with survival post-chemotherapy. Insufficient data was available in assessing LKB1 expression survival associations pre-chemotherapy, however higher expression of LKB1 was significantly predictive of improved survival in the post-chemotherapy group. CAB39, by contrast, was predictive of worse survival post-chemotherapy (Table 1).

#### HER2-enriched

In HER2-enriched breast cancer, higher NUAK1 expression was associated with worse patient survival in both pre- and post-chemotherapy groups. In the post-chemotherapy group, MARK1, MARK2, SNRK, and QSK expression was significantly associated with lower survival (Table 1).

#### Basal-like

In Basal-like breast cancer, both MARK1 and MARK3 were positively associated with patient survival in the pre-chemotherapy group but negatively associated with survival post-chemotherapy. In this subtype, high LKB1 and NUAK1 expression was predictive of lower survival outcomes in the post-chemotherapy group, with no significant difference in the pre-chemotherapy group (Table 1).

#### ER +

In ER+ breast cancer, QSK mRNA gene expression showed similar prognosis in survivability in systemically treated and untreated cohorts. Although we were unable to obtain data for BRSK2 survival pre- chemotherapy, higher BRSK2 expression was correlated to improved survival rates post treatment (Table 2).

**Table 2:** Hazard ratios (HR) and associated confidence intervals (CI) comparing survival with and without systemic chemotherapy as a function of mRNA gene expression of select LKB1 downstream kinases in IHC-based breast cancer subtypes using the Kaplan–Meier estimator

		HR (CI)		*P* Value	
	Gene	No Treatment	Systemic Treatment	No Treatment	Systemic Treatment
**TNBC**	**PAK1 (226507)**	*0.26 (0.07–.097)*	0.51 (0.25–1.05)	0.032	0.063
	**SIK2**	*0.18 (0.04–0.8)*	0.54 (0.27–1.11)	0.011	0.0903
**ER +**	**QSK**	*0.55 (0.41–0.74)*	*0.64 (0.49–0.84)*	0.000069	0.0011
	**BRSK2**	-	*0.76 (0.58–0.99)*	-	0.041
**Mutant TP53**	**BRSK1**	-	*0.23 -(.06–0.82)*	-	0.013

HR and associated CI in Italic indicate indicate positive survival effect with statistical significance of *p* < 0.05. Some genes are not shown either due to insufficient or non-significant data for both treatment conditions.

#### Mutant TP53

In mutant TP53 breast cancer, pre- chemotherapy data for BRSK1 did not exist. However, patient survivability was favorable post treatment with increased BRSK1 mRNA expression (Table 2).

#### TNBC

In TNBC, increased expression of PAK1 and SIK2 showed better survivability in pre-chemotherapy groups. There was no statistical significance post-treatment (Table 2).

## DISCUSSION

Based on our data analysis, LKB1 signaling as measured by levels of mRNA expression of immediate downstream targets had variable outcomes on patient survival depending on the breast cancer subtype. In all breast cancer combined, high expression of LKB1, AMPK, LYK5, MARK1, MARK2, NUAK2, PAK1 (both probe sets), SIK1, SIK2, BRSK1, BRSK2, SNRK, and QSK was positively correlated with improved survival compared to low expression of these genes. The remaining genes, CAB39, MARK3, MARK4, and NUAK1 had an insignificant statistical correlation. Additional breast cancer subtype analyses revealed discrepancies when individual LKB1 downstream targets and LKB1 alone were examined. For instance, NUAK2 expression was positively correlated with survival in overall, TNBC, ER-, and all intrinsic molecular subtypes, but negatively associated with survival in ER+ and WTp53, and had no correlation in mutant p53 breast cancer. Interestingly, PAK1 expression and QSK expression are consistently associated with positive survival outcomes in all subtypes with the exceptions of WT P53 and ER- subtypes.

In groups stratified to include chemotherapy treated and untreated breast cancer cases, TNBC patients with high expressions of PAK1 (226507) and SIK2, ER+ patients with high expression of QSK and BRSK2, and mutant TP53 patients with high BRSK1 expression all had an improved survival correlation post chemotherapy. However, there was insufficient data available to compare BRSK2 in ER+ and BRSK1 in mutant TP53 to untreated groups, warranting further investigation in order to properly correlate expression levels and chemotherapy. There are also notable deviations from gene-specific survival associations in each intrinsic molecular subtype once treatment status is considered. In Luminal A breast cancers, the positive survival effects of AMPK, MARK3, PAK1, BRSK1, SNRK, and QSK are maintained in the pre-chemotherapy but not in the post-chemotherapy cohorts. Notably, LYK5 expression is negatively associated with survival in the post-chemotherapy setting, whereas it had a positive survival association in overall Luminal A. In the Luminal B subtype, high MARK2 expression was noted to be negatively associated with survival in pre- and post-chemotherapy groups. Conversely, MARK2 had no significant effect on survival in the overall Luminal B subtype, and a significantly positive effect in the ER+ subtype, a subtype which overlaps clinicopathologically with Luminal B [8]. This provides a basis for using ER expression as a biomarker for studying MARK2 activity in a basic science setting.

Several genes exhibited a shift from a positive effect of expression on survival pre-chemotherapy to a negative association with survival post-chemotherapy. These include SIK1 in Luminal B and MARK1 and MARK3 in the Basal-like subtype. Similarly, in overall breast cancers exposed to systemic chemotherapy we found that high expression of AMPK, SIK2, and QSK led to increases in hazard ratios compared to untreated. These trends suggest that chemotherapy may select for highly aggressive forms of disease where the protective effects of these particular genes are diminished.

In addition to the KMPlot results presented thus far, we have generated baseline LKB1 signaling data in various breast cancer cell lines and patient derived xenograft (PDX) models (Supplementary Figures 1–3). This data provides supporting information comparing and contrasting relevant established cell lines and PDX models to generate a platform for other researchers’ investigations in LKB1 signaling. For example, those interested in studying BRSK2 activity in breast cancer may be inclined to use MCF7 cells due to higher levels seen by qPCR and immunoblot (Supplementary Figures 1 and 2). In addition to cell lines, we have immunoblotting data for characterized PDX models. For example, TU-BcX-4IC’s would be an ideal model for studying the effects of low LKB1 activity in a more aggressive and drug-resistant disease (Supplementary Figure 3) [9].

In conclusion, these data demonstrate LKB1 and its downstream targets are differently correlated with patient survival, depending on subtype and chemotherapy exposure. These findings support the rationale for additional in-depth studies to elucidate the role of LKB1 signaling, including detailed studies on those targets LKB1 phosphorylates, in breast cancer development in specific molecular subtypes in order to improve clinical outcomes.

## SUPPLEMENTARY MATERIALS







## References

[1] Bonanno L , Zulato E , Pavan A , Attili I , Pasello G , Conte P , Indraccolo S . LKB1 and Tumor Metabolism: The Interplay of Immune and Angiogenic Microenvironment in Lung Cancer. Int J Mol Sci. 2019; 20:1874. 10.3390/ijms20081874. 30995715 PMC6514929

[2] Shackelford DB , Shaw RJ . The LKB1-AMPK pathway: metabolism and growth control in tumour suppression. Nat Rev Cancer. 2009; 9:563–75. 10.1038/nrc2676. 19629071 PMC2756045

[3] Rhodes LV , Tate CR , Hoang VT , Burks HE , Gilliam D , Martin EC , Elliott S , Miller DB , Buechlein A , Rusch D , Tang H , Nephew KP , Burow ME , Collins-Burow BM . Regulation of triple-negative breast cancer cell metastasis by the tumor-suppressor liver kinase B1. Oncogenesis. 2015; 4:e168. 10.1038/oncsis.2015.27. 26436950 PMC4632088

[4] Ciccarese F , Zulato E , Indraccolo S . LKB1/AMPK Pathway and Drug Response in Cancer: A Therapeutic Perspective. Oxid Med Cell Longev. 2019; 2019:8730816. 10.1155/2019/8730816. 31781355 PMC6874879

[5] Makeld T , Monni O , Vainio S . LKB 1 tumor suppression in Peutz-Jeghers Syndrome. 2008.

[6] Györffy B , Lanczky A , Eklund AC , Denkert C , Budczies J , Li Q , Szallasi Z . An online survival analysis tool to rapidly assess the effect of 22,277 genes on breast cancer prognosis using microarray data of 1,809 patients. Breast Cancer Res Treat. 2010; 123:725–31. 10.1007/s10549-009-0674-9. 20020197

[7] Li Q , Birkbak NJ , Gyorffy B , Szallasi Z , Eklund AC . Jetset: selecting the optimal microarray probe set to represent a gene. BMC Bioinformatics. 2011; 12:474. 10.1186/1471-2105-12-474. 22172014 PMC3266307

[8] Goldhirsch A , Winer EP , Coates AS , Gelber RD , Piccart-Gebhart M , Thürlimann B , Senn HJ , Panel M , and Panel members. Personalizing the treatment of women with early breast cancer: highlights of the St Gallen International Expert Consensus on the Primary Therapy of Early Breast Cancer 2013. Ann Oncol. 2013; 24:2206–23. 10.1093/annonc/mdt303. 23917950 PMC3755334

[9] Chang TC , Matossian MD , Elliott S , Burks HE , Sabol RA , Ucar DA , Wathieu H , Zabaleta J , Valle L , Gill S , Martin E , Riker AI , Miele L , et al. Evaluation of deacetylase inhibition in metaplastic breast carcinoma using multiple derivations of preclinical models of a new patient-derived tumor. PLoS One. 2020; 15:e0226464. 10.1371/journal.pone.0226464. 33035223 PMC7546483

