# Supplementary material for: Evaluation of liver kinase B1 downstream signaling expression in various breast cancers and relapse free survival after systemic chemotherapy treatment
Source: Oncotarget. 2021 May 25;12(11):1110–5. doi: 10.18632/oncotarget.27929 (PMC8169068; doi:10.18632/oncotarget.27929)
Supplement: Supplementary file 1 [file oncotarget-12-1110-s001.pdf]

Evaluation of liver kinase B1 downstream signaling expression in various breast cancers and relapse free survival after systemic chemotherapy treatment

SUPPLEMENTARY MATERIALS

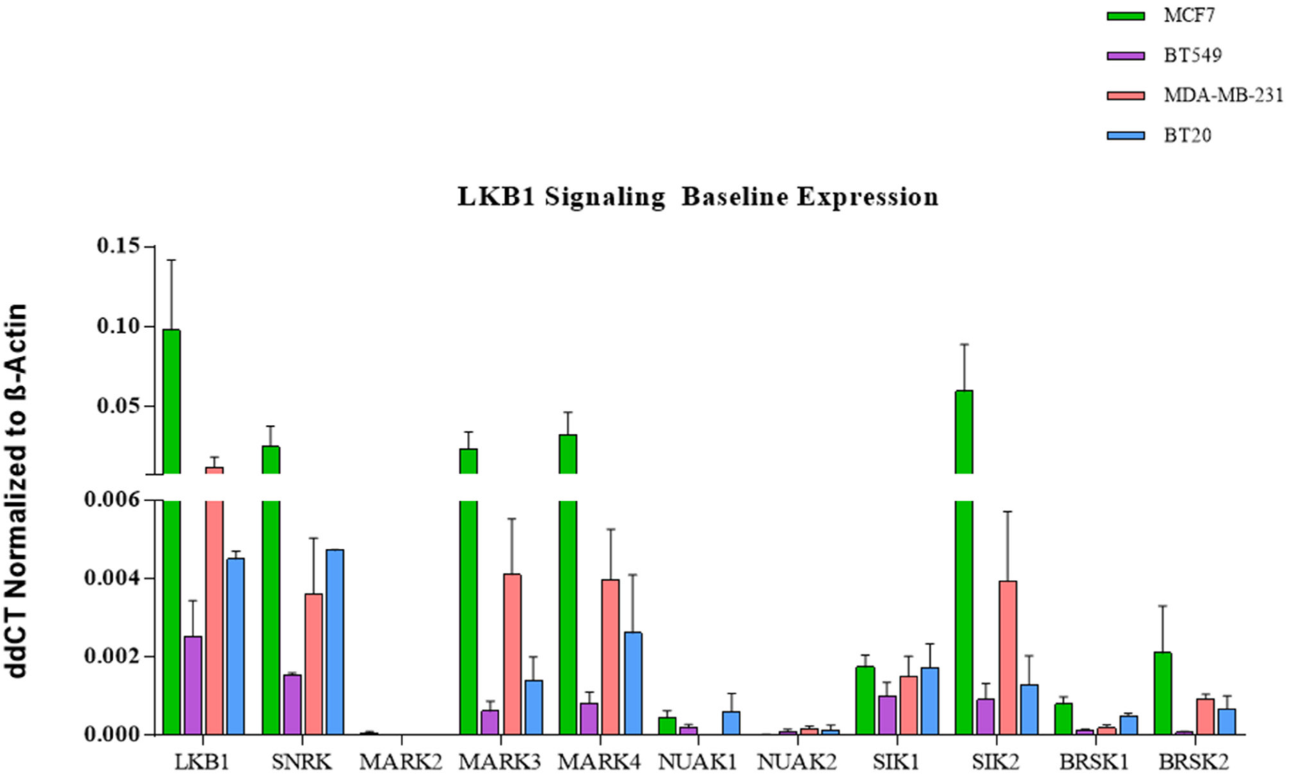

Supplementary Figure 1: Cells were cultured in media as recommended by ATCC and harvested between 80–90% confluency. qPCR analysis of baseline signaling for breast cancer cell lines. SNRK is an upstream regulator of LKB1 signaling. Values are normalized to ß-actin levels.

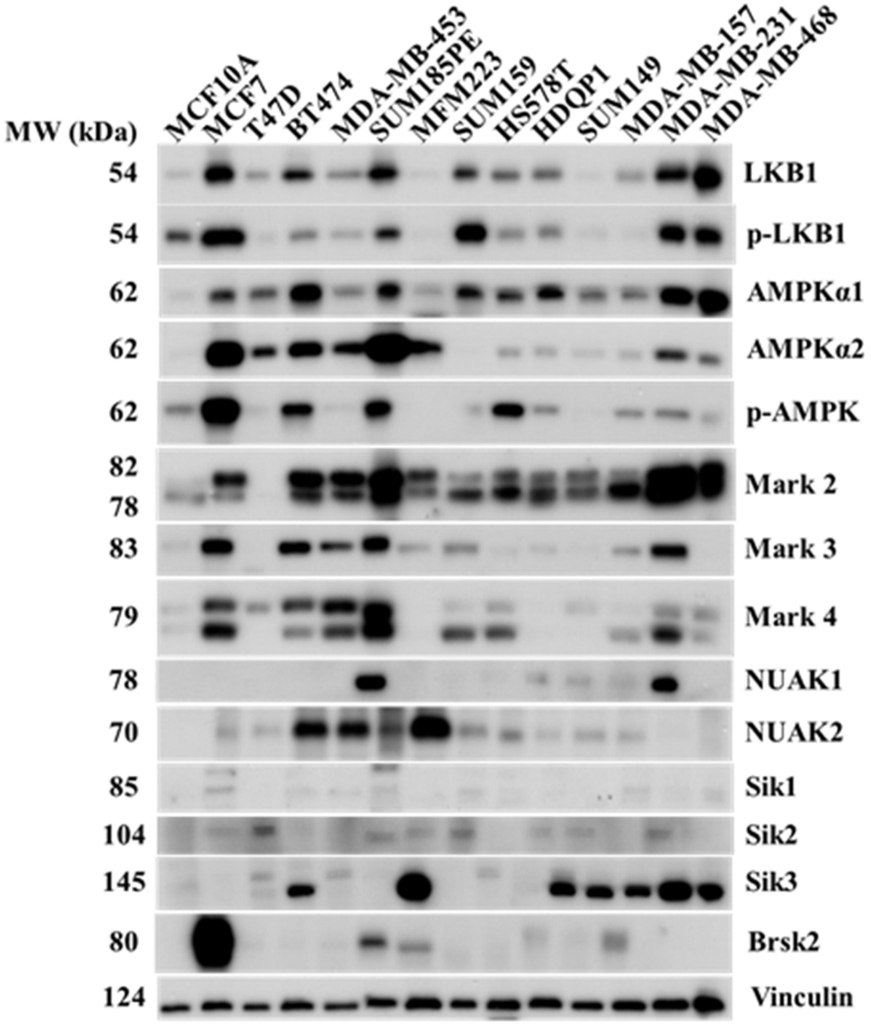

Supplementary Figure 2: Cells were cultured in media as recommended by ATCC and harvested between 80–90% confluency. Immunoblot analysis of baseline signaling for breast cancer cell lines. Vinculin was used as a loading control.

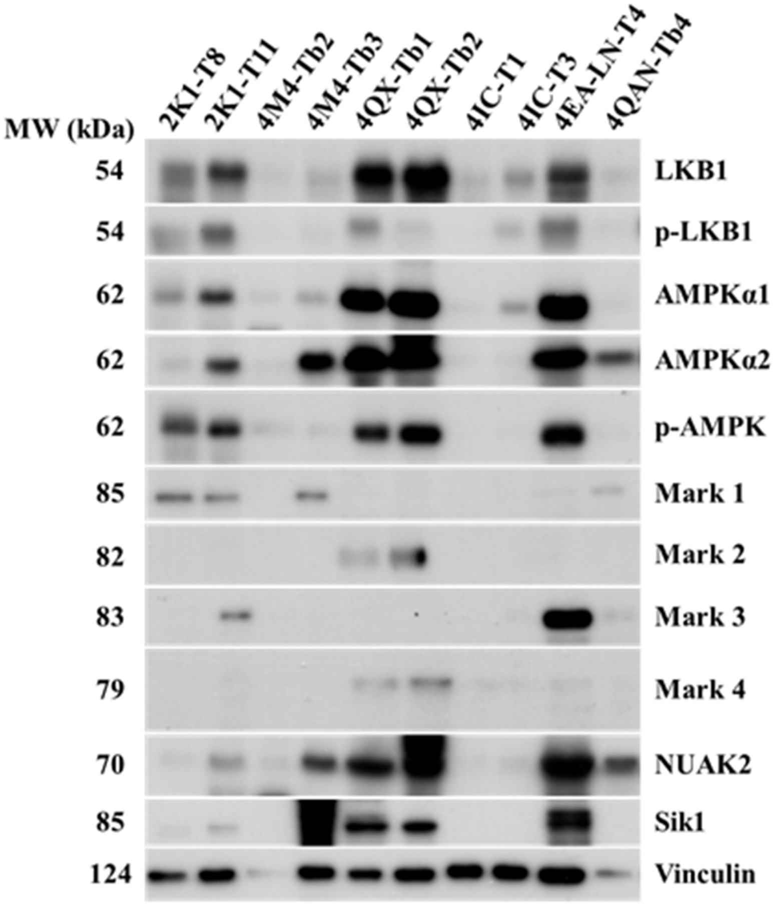

Supplementary Figure 3: PDX models were generated by the Burow Lab in collaboration with Tulane hospital, UMC, and the LCRC. T refers to transplant passage number, the letter after the T represents site of tumor (e.g. Ta and Tb would denote xenografts generated from same patient but different tumor sites), and LN denotes tumor taken from lymph nodes. Vinculin was used as a loading control.

Supplementary Table 1: Hazard ratios (HR) and associated confidence intervals (CI) for patient survival as a function of mRNA gene expression of select LKB1 downstream kinases in all breast cancer and intrinsic subtypes using the Kaplan–Meier estimator. See Supplementary Table 1

Supplementary Table 2: Hazard ratios (HR) and associated confidence intervals (CI) for patient survival as a function of mRNA gene expression of select LKB1 downstream kinases in IHC-based breast cancer subtypes using the Kaplan–Meier estimator using the Kaplan–Meier estimator. See Supplementary Table 2
