# Supplementary material for: Evaluation of liver kinase B1 downstream signaling expression in various breast cancers and relapse free survival after systemic chemotherapy treatment
Source: Oncotarget. 2021 May 25;12(11):1110–5. doi: 10.18632/oncotarget.27929 (PMC8169068; doi:10.18632/oncotarget.27929)
Supplement: Supplementary file 2 [file oncotarget-12-1110-s002.docx]

**Supplementary Table 1: Hazard ratios (HR) and associated confidence intervals (CI) for patient survival as a function of mRNA gene expression of select LKB1 downstream kinases in all breast cancer and intrinsic subtypes using the Kaplan–Meier estimator**

|  | **All Breast Cancer** | | **Luminal A** | | **Luminal B** | | **HER2-enriched** | | **Basal-like** | |
| --- | --- | --- | --- | --- | --- | --- | --- | --- | --- | --- |
| **Gene** | **HR (CI)** | **P Value** | **HR (CI)** | **P Value** | **HR (CI)** | **P Value** | **HR (CI)** | **P Value** | **HR (CI)** | **P Value** |
| **LKB1** (231017_at) | 0.67 (0.57-0.76) | 0.00000056 | 0.62(0.48 − 0.8) | 0.00023 | 0.67 (0.49 − 0.91) | 0.0094 | - | - | 1.21 (0.83 − 1.76) | 0.33 |
| **AMPK** (207709_at) | 0.74 (0.65-0.84) | 0.0000023 | 0.75(0.63 − 0.89) | 0.0011 | 0.58 (0.46 − 0.73) | 0.0000024 | - | - | 0.81 (0.63 − 1.05) | 0.11 |
| **CAB39** (217873_at) | 1.11 (0.99-1.24) | 0.072 | 1.14(0.96 − 1.35) | 0.14 | 1.14 (0.96 − 1.35) | 0.14 | - | - | 0.76 (0.58 − 1) | 0.046 |
| **LYK5** (52169_at) | 0.51 (0.45-0.57) | 1E-16 | 0.51(0.43 − 0.61) | 4.7E-14 | 0.55 (0.45 − 0.67) | 2.8E-09 | 0.61 (0.4 − 0.91) | 0.014 | 0.43 (0.33 − 0.55) | 5.1E-11 |
| **MARK1** (226653_at) | 0.81 (0.7-0.95) | 0.011 | 0.68(0.52 − 0.88) | 0.0036 | 0.76 (0.56 − 1.04) | 0.084 | 0.61 (0.38 − 0.96) | 0.03 | 0.69 (0.49 − 0.96) | 0.029 |
| **MARK2** (203942_at) | 0.76 (0.68-0.85) | 0.0000011 | 0.65(0.55 − 0.77) | 0.00000094 | 0.88 (0.73 − 1.07) | 0.19 | - | - | 0.64 (0.5 − 0.82) | 0.00046 |
| **MARK3** (22569_s_at) | 1.11 (1-1.24) | 0.054 | 0.87(0.72 − 1.05) | 0.15 | 1.24 (1.02 − 1.51) | 0.03 | - | - | 1.29 (0.98 − 1.71) | 0.069 |
| **MARK4** (221560_at) | 1.08 (0.97-1.21) | 0.16 | 1.23(1.04 − 1.46) | 0.015 | 0.82 (0.68 − 1) | 0.054 | 0.67 (0.45 − 0.99) | 0.044 | 0.85 (0.66 − 1.09) | 0.21 |
| **NUAK1** (204589_at) | 1.1 (0.98-1.23) | 0.098 | 1.13(0.93 − 1.37) | 0.21 | 1.54 (1.23 − 1.93) | 0.00016 | 1.85 (1.2 − 2.85) | 0.0047 | 1.38 (1.05 − 1.81) | 0.018 |
| **NUAK2** (220987_s_at) | 0.56 (0.5-0.63) | 1E-16 | 0.63(0.53 − 0.75) | 0.00000011 | 0.78 (0.63 − 0.97) | 0.023 | 0.27 (0.15 − 0.47) | 0.00000079 | 0.44 (0.34 − 0.58) | 6.2E-10 |
| **PAK1** (202161) | 0.75 (0.66-0.84) | 0.0000017 | 0.73(0.61 − 0.87) | 0.00052 | 1.02 (0.84 − 1.23) | 0.88 | 0.59 (0.4 − 0.86) | 0.0058 | 0.68 (0.53 − 0.88) | 0.0031 |
| **PAK1** (226507) | 0.54 (0.46-0.64) | 5.6E-14 | 0.67(0.52 − 0.86) | 0.0014 | 0.47 (0.35 − 0.64) | 0.00000072 | 0.53 (0.33 − 0.85) | 0.0083 | 0.54 (0.39 − 0.75) | 0.00015 |
| **SIK1** (208078_s_at) | 0.77 (0.69-0.86) | 0.0000019 | 0.71(0.58 − 0.86) | 0.00051 | 0.67 (0.54 − 0.82) | 0.00008 | - | - | 0.72 (0.56 − 0.94) | 0.014 |
| **SIK2** (1556056_at) | 0.67 (0.57-0.78) | 0.00000027 | 0.62(0.48 − 0.79) | 0.00011 | 0.66 (0.49 − 0.9) | 0.0087 | - | - | 0.48 (0.33 − 0.69) | 0.000067 |
| **BRSK1** (1552504_a_at) | 0.73 (0.62-0.86) | 0.000097 | 0.77(0.6 − 0.98) | 0.036 | 0.63 (0.44 − 0.91) | 0.012 | 0.42 (0.26 − 0.69) | 0.00038 | 0.39 (0.28 − 0.54) | 3.2E-09 |
| **BRSK2** (223715_at) | 0.66 (0.57-0.78) | 0.00000043 | 0.69(0.54 − 0.9) | 0.0055 | 0.76 (0.55 − 1.06) | 0.11 | 0.55 (0.34 − 0.89) | 0.013 | 0.6 (0.43 − 0.83) | 0.0017 |
| **SNRK** (209481_at) | 0.64 (0.57-0.73) | 1.3E-12 | 0.6(0.5 − 0.73) | 0.00000011 | 0.74 (0.61 − 0.9) | 0.002 | - | - | 0.78 (0.6 − 1.01) | 0.055 |
| **QSK** (213034_at) | 0.55 (0.49-0.62) | 1E-16 | 0.45(0.38 − 0.53) | 1E-16 | 0.58 (0.48 − 0.71) | 0.000000072 | 0.55 (0.37 − 0.82) | 0.0025 | 0.68 (0.52 − 0.87) | 0.0027 |

HR and associated CI with statistical significance of p < 0.05 are colored in blue to indicate positive survival effect, while those colored in red indicate negative survival effect. (-) indicates insufficient data.
