# Supplementary material for: Evaluation of liver kinase B1 downstream signaling expression in various breast cancers and relapse free survival after systemic chemotherapy treatment
Source: Oncotarget. 2021 May 25;12(11):1110–5. doi: 10.18632/oncotarget.27929 (PMC8169068; doi:10.18632/oncotarget.27929)
Supplement: Supplementary file 3 [file oncotarget-12-1110-s003.docx]

**Supplementary Table 2: Hazard ratios (HR) and associated confidence intervals (CI) for patient survival as a function of mRNA gene expression of select LKB1 downstream kinases in IHC-based breast cancer subtypes using the Kaplan–Meier estimator using the Kaplan–Meier estimator**

|  | **ER-** | | **ER+** | | **WT Tp53** | | **Mutant Tp53** | | **TNBC** | |
| --- | --- | --- | --- | --- | --- | --- | --- | --- | --- | --- |
| **Gene** | **HR (CI)** | **P Value** | **HR (CI)** | **P Value** | **HR (CI)** | **P Value** | **HR (CI)** | **P Value** | **HR (CI)** | **P Value** |
| **LKB1** (231017_at) | 1.56 (1.12-2.17) | 0.0081 | 0.88 (0.66-1.19) | 0.41 | 1.94 (0.81-4.64) | 0.13 | 0.45 (0.25-0.82) | 0.0071 | 1.66 (0.93-2.97) | 0.083 |
| **AMPK** (207709_at) | 0.86 (0.68-1.07) | 0.18 | 0.84 (0.71-1) | 0.055 | 0.65 (0.43-1) | 0.048 | 1.55 (0.95-2.54) | 0.077 | 1.36 (0.86-2.16) | 0.19 |
| **CAB39** (217873_at) | 1.27(1.01-1.59) | 0.039 | 1.14 (0.97-1.34) | 0.12 | 0.78 (0.5-1.22) | 0.28 | 0.62 (0.38-1.04) | 0.066 | 1.35 (0.89-2.07) | 0.16 |
| **LYK5** (52169_at) | 1.2 (0.94-1.52) | 0.14 | 0.84 (0.7-1) | 0.056 | 1.58 (1.03-2.42) | 0.033 | 0.7 (0.43-1.14) | 0.15 | 1.43 (0.94-2.19) | 0.094 |
| **MARK1** (226653_at) | 1.71 (1.11-2.62) | 0.013 | 1.45 (1.06-1.98) | 0.02 | 2.35 (0.78-7.08) | 0.12 | 0.59 (0.29-1.19) | 0.14 | 0.7 (0.4-1.2) | 0.19 |
| **MARK2** (203942_at) | 1.17 (0.93-1.46) | 0.18 | 0.76 (0.65-0.93) | 0.0052 | 1.7 (1.12-2.6) | 0.012 | 1.35 (0.83-2.19) | 0.22 | 0.74 (0.45-1.22) | 0.24 |
| **MARK3** (22569_s_at) | 1.3 (1.01-1.68) | 0.038 | 0.87 (0.74-1.3) | 0.1 | 0.61 (0.39-0.95) | 0.027 | 1.49 (0.81-2.72) | 0.2 | 1.58 (1.01-2.45) | 0.041 |
| **MARK4** (221560_at) | 0.88 (0.69-1.14) | 0.34 | 0.86 (0.72-1.03) | 0.1 | 1.77 (1.13-2.75) | 0.011 | 0.55 (0.34-0.89) | 0.014 | 1.31 (0.62-2.08) | 0.26 |
| **NUAK1** (204589_at) | 1.73 (1.38-2.18) | 0.0000015 | 1.28 (1.04-1.57) | 0.017 | 1.46 (0.96-2.22) | 0.078 | 1.5 (0.81-2.81) | 0.2 | 1.84 (1.18-2.87) | 0.0061 |
| **NUAK2** (220987_s_at) | 0.7 (0.55-0.89) | 0.0039 | 1.22 (1.03-1.44) | 0.02 | 1.7 (1.12-2.6) | 0.013 | 0.68 (0.42-1.13) | 0.13 | 0.58 (0.38-0.89) | 0.011 |
| **PAK1** (202161) | 0.7 (0.55-0.89) | 0.003 | 1.16 (0.97-1.39) | 0.11 | 1.58 (1.02-2.45) | 0.04 | 1.49 (0.83-2.69) | 0.18 | 0.55 (0.36-0.84) | 0.0054 |
| **PAK1** (226507) | 0.59 (0.42-0.82) | 0.0019 | 0.71 (0.53-0.96) | 0.026 | 0.75 (0.29-1.92) | 0.55 | 0.27 (0.15-0.48) | 0.0000021 | 0.44 (0.26-0.76) | 0.0027 |
| **SIK1** (208078_s_at) | 0.85 (0.66-1.08) | 0.18 | 0.8 (0.66-0.96) | 0.019 | 1.28 (0.82-2) | 0.28 | 0.57 (0.35-0.92) | 0.02 | 0.77 (0.69-0.86) | 0.0000019 |
| **SIK2** (1556056_at) | 0.75 (0.53-1.07) | 0.11 | 0.75 (0.55-1.02) | 0.065 | 0.65 (0.26-1.58) | 0.34 | 1.91 (1.06-3.43) | 0.029 | 0.46 (0.22-0.98) | 0.038 |
| **BRSK1** (1552504_a_at) | 0.62 (0.44-0.88) | 0.0069 | 1.23 (0.91-1.65) | 0.18 | 1.75 (0.73-4.17) | 0.2 | 0.2 (0.09-0.45) | 0.000017 | 0.6 (0.33-1.09) | 0.092 |
| **BRSK2** (223715_at) | 1.4 (0.99-2) | 0.059 | 1.23 (0.91-1.67) | 0.18 | 0.57 (0.23-1.4) | 0.21 | 1.44 (0.78-2.68) | 0.24 | 0.64 (0.36-1.14) | 0.13 |
| **SNRK** (209481_at) | 0.89 (0.69-1.15) | 0.37 | 0.79 (0.67-0.93) | 0.0057 | 0.73 (0.48-1.12) | 0.15 | 2.34 (1.16-4.72) | 0.014 | 0.62 (0.38-1.02) | 0.057 |
| **QSK** (213034_at) | 0.82 (0.62-1.08) | 0.16 | 0.59 (0.5-0.7) | 1.9E-10 | 0.55 (0.35-0.86) | 0.0075 | 0.51 (0.31-0.84) | 0.0075 | 0.4 (0.22-0.73) | 0.0018 |

HR and associated CI with statistical significance of p < 0.05 are colored in blue to indicate positive survival effect, while those colored in red indicate negative survival effect. (-) indicates insufficient data.
